# Synthesis of Three Isoelemental MXenes and Their Structure–Property Relationships

**DOI:** 10.1021/jacs.4c11111

**Published:** 2024-10-31

**Authors:** Marley Downes, Christopher E. Shuck, Ruocun John Wang, Paweł Piotr Michałowski, Jonathan Shochat, Danzhen Zhang, Mikhail Shekhirev, Yizhou Yang, Nestor J. Zaluzec, Raul Arenal, Steven J. May, Yury Gogotsi

**Affiliations:** 1 A. J. Drexel Nanomaterials Institute, and Department of Materials Science and Engineering, Drexel University, Philadelphia, Pennsylvania 19104, United States; 2 Department of Chemistry and Chemical Biology, Rutgers University, Piscataway, New Jersey 08854, United States; 3 Łukasiewicz Research Network—Institute of Microelectronics and Photonics, Warsaw 02-668, Poland; 4 Pritzker School of Molecular Engineering, Laboratory for Energy Storage and Conversion, Chicago, and Argonne National Laboratory, University of Chicago, Lemont, Illinois 60637, United States; 5 Instituto de Nanociencia y Materiales de Aragon (INMA), CSIC-Universidad de Zaragoza, Zaragoza 50018, Spain; 6 Laboratorio de Microscopias Avanzadas (LMA), Universidad de Zaragoza, Zaragoza 50018, Spain; 7 ARAID Foundation, Zaragoza 50018, Spain

## Abstract

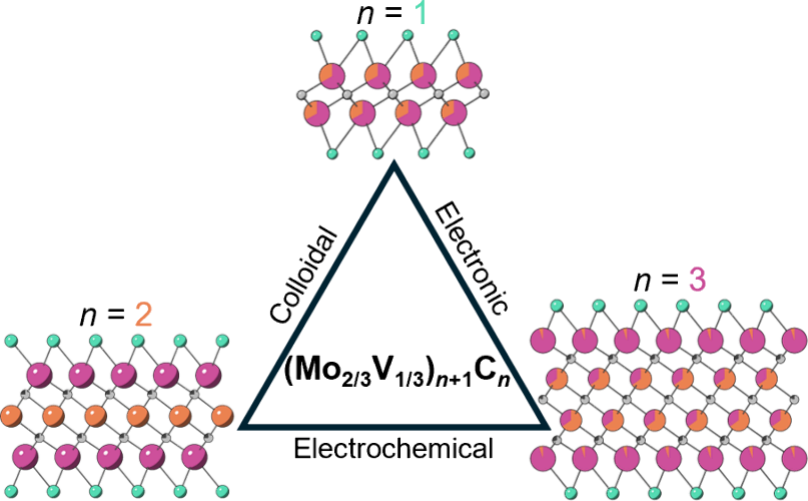

The MXene family has rapidly expanded since its discovery in 2011 to include nearly 50 unique MXenes, not accounting for solid solutions and diverse surface terminations. However, a question raised since their discovery has been: What is the effect of *n*? In other words, how does the number of layers affect the MXene properties? To date, no direct study of the impact of *n* has been conducted due to the lack of isoelemental MXene compositions spanning more than two *n* values. Herein, we report on a system of three MXenes with identical M-site chemistries, (Mo_2/3_V_1/3_)_*n*+1_C_*n*_T_*x*_ (*n* = 1, 2, and 3), allowing for the study of MXene structure–property relationships across *n*, for the first time. Chemical analysis of the samples shows complete and partial ordering of the M-elements in the *n* = 2 and 3 samples, respectively. We show that sample stability gradually evolves as *n* is increased from 1 to 3, while electronic and electrochemical properties exhibit more significant changes in going from *n* = 1 to 2 than from *n* = 2 to 3.

## Introduction

MXenes are a large class of two-dimensional (2D) transition metal carbides, nitrides, and carbonitrides with the general formula of M_*n*+1_X_*n*_T_*x*_, where M is an early transition metal (Ti, V, Mo, Nb, etc.), X is C and/or N, T_*x*_ represents the surface groups (typically −O, −OH, −F, and −Cl, but will not be continually expressed for clarity), and *n* is an integer 1–4 that represents the number of X-site atomic layers.^1,2^ Because MXenes are readily scalable,^3,4^ easily processable,^5−7^ and have highly desirable optical,^8−13^ electrical,^9,12,14−16^ and mechanical properties,^16−19^ they have been widely explored in fields such as electrochemical energy storage,^8,20−23^ electromagnetic interference (EMI) shielding,^24−26^ and biomedicine.^27−29^ Nearly 50 stoichiometric MXenes have been synthesized to date, including multiple structures, such as ordered double transition metal MXenes,^30,31^ in-plane ordered MXenes,^32−34^ high-entropy MXenes,^35,36^ and solid-solution MXenes,^2,12,37−39^ illustrating the variety of MXene structures that already exist. There are also numerous solid solutions on M and X sites, as well as MXenes with diverse surface terminations reported. However, considering how diverse the MXene family is, a question that has been posed since MXenes were first discovered remains unanswered: how does *n* affect MXene properties?

Experimentally, this poses a challenge: Ti_2_C and Ti_3_C_2_ exist, but not Ti_4_C_3_; Nb_2_C and Nb_4_C_3_, but not Nb_3_C_2_; Mo_2_TiC_2_ and Mo_2_Ti_2_C_3_, but not (MoTi)_2_C. This trend has been repeated across all MXene chemistries discovered thus far, where no series of MXene compositions with the same transition metals currently exist. Despite the lack of a directly comparable sample set, there have been numerous attempts to study the impact of *n* on different functional properties. For instance, increasing *n* increases the thermal stability of the MXene. This trend has been observed across M—with Ti_3_C_2_ having higher thermal stability than Nb_2_C and Mo_2_C.^40^ Thicker MXenes have also shown increased colloidal stability over their thinner counterparts, as shown by the higher oxidative stability of Ti_3_C_2_ over Ti_2_C, and Nb_4_C_3_ over Nb_2_C.^41,42^ However, many of the approaches used to improve the stability of Ti_3_C_2_, including cooling, bubbling with inert gas, minimizing the volume of headspace in the bottle, and others, can also be applied to improve the stability of Ti_2_C.^41^ It is not easy to extrapolate and make predictions based on 2 data points. Thus, further studies are needed to understand the direct influence of *n* on the atomic structure of MXenes (e.g., Ti and C vacancy formation energies) as well as the physicochemical behavior of MXene nanosheets (e.g., surface acidity, charge, and zeta potential).

Once again, when the application-driven properties, are examined, a strong dependence on *n* is observed. In terms of the mechanical properties, increasing *n* will result in increased rigidity and mechanical strength.^17,18,43^ Higher *n* MXenes have been shown to exhibit higher electronic conductivity and, thus, higher electromagnetic interference (EMI) shielding in a broad frequency range, making them more promising for EMI shielding when normalized by the film thickness.^25^ The optical properties of MXenes have shown a dependency on both M and *n*, with blue shifts of the wavelength of the absorption peak thought to occur with increasing free carrier concentration of the MXene.^11^ Even considering these studies, experimental determination of the effect of *n* is severely limited, and with only two points, it is difficult to determine any trends. Additionally, many studies focus only on Ti_3_C_2_ and its derivatives, largely due to its greatly improved synthesis and popularity across research fields. Computationally, there have been several studies focused on this issue; it is much easier to vary *n* in theoretical models than in experimentally. Many of these studies have shown that the MXene thickness significantly affects their electronic properties.^44,45^ There have also been theoretical studies on the mechanical properties,^43,46^ thermal stability,^47^ oxidative stability,^48^ and others.^49^ However, experimental validation of these results has been limited due to MXene availability and the differing synthesis methods utilized across research groups, preventing true comparisons of results.

Herein, we use the (Mo_2/3_V_1/3_)_*n*+1_C_*n*_ (*n* = 1, 2, 3) system as a model to study the effect of *n* on MXene properties. The syntheses of each MAX phase, (Mo_2/3_V_1/3_)_2_AlC, (Mo_2/3_V_1/3_)_3_AlC_2_, and (Mo_2/3_V_1/3_)_4_AlC_3_ were synthesized with no other MAX impurities, followed by topochemical MXene synthesis and delamination to produce the corresponding (Mo_2/3_V_1/3_)_2_C, (Mo_2/3_V_1/3_)_3_C_2_, and (Mo_2/3_V_1/3_)_4_C_3_. We provide structural and chemical characterizations of these materials using high-resolution scanning transmission electron microscopy (HRSTEM) imaging, STEM energy-dispersive X-ray spectroscopy (STEM-EDS), X-ray diffraction (XRD), and secondary ion mass spectrometry (SIMS) and explore their colloidal, optical, and electronic properties. Finally, the electrochemical behavior of these MXenes is studied to gain insights into their accessible voltage windows, cycling stability, and capacitance.

## Experimental Section

### Synthesis of (Mo_2/3_V_1/3_)_*n*+1_AlC_*n*_ MAX Phases

Molybdenum (Thermo Fisher Scientific, ∼250 mesh, 99.9% metals basis), vanadium (Thermo Fisher Scientific, ∼325 mesh, 99.5%), vanadium(III) oxide (Alfa Aesar, 99.2%), aluminum (Thermo Fisher Scientific, ∼325 mesh, 99.5% (metals basis)), and graphite (Alfa Aesar, ∼325 mesh, 99%) powders were combined in the appropriate atomic ratios. For all powders, the Mo:V ratio was held constant at 2/3:1/3. For (Mo_2/3_V_1/3_)_2_AlC the powders were mixed in a MoV:Al:C = 2:1.1:0.9 molar ratio; for (Mo_2/3_V_1/3_)_3_AlC_2_ the ratio was MoV:Al:C = 2:1:1.1:1.8 + 5% V_2_O_3_; and for (Mo_2/3_V_1/3_)_4_AlC_3_ the ratio was MoV:Al:C = 4:1.1:2.7. After mixing the powders were ball-milled at 70 rpm for 12 h at a ratio of 1:2 = powder:yttria-stabilized zirconia (YSZ) balls by mass. The mixtures were then heated in alumina crucibles at a 3 °C min^–1^ ramp (the same rate was used for cooling) under 350 cm^3^ min^–1^ flowing argon in a tube furnace (Carbolite Gero). To synthesize (Mo_2/3_V_1/3_)_2_AlC and (Mo_2/3_V_1/3_)_3_AlC_2_, the powders were held at 1550 °C for 2 h. To synthesize (Mo_2/3_V_1/3_)_4_AlC_3_, the powders were held at 1600 °C for 4 h.

The sintered materials were then ground to powder using a combination of CNC mill and mortar and pestle. The resulting MAX-phases were stirred in 9 M HCl for 24 h to dissolve intermetallic impurities, then washed to neutral pH by vacuum filtration with water. The powders were collected and dried overnight at room temperature under vacuum. The powders were then sieved using stainless-steel meshes to obtain a particle size of <45 μm for uniform etching.

### Synthesis of (Mo_2/3_V_1/3_)_*n*+1_C_*n*_ MXenes

For uniformity in the synthesis method, all of the studied MAX phases were etched via the same method. For the synthesis of multilayer MXenes, 5 g of HCl-washed MAX phase powders were slowly added to a solution of 60 mL of HF (48–51%, Arcos Organics), 60 mL of HCl (12 M, Fisher Chemical), and 30 mL of deionized water while being stirred with a polytetrafluoroethylene (PTFE)-coated stir bar at 300 rpm. The reaction proceeded for 5 days at 35 °C. After the etching reaction completion, the samples were washed via a series of cycles involving centrifugation at 3500 rpm (2550 rcf) for 10 min, decanting the acidic supernatant, and redispersion of the sediment using deionized (DI) water until the supernatant reached a neutral pH.

The multilayer MXene powder was then collected and used to make a 5 wt % tetramethylammonium hydroxide solution (TMAOH, 25 wt % Sigma-Aldrich). The delamination intercalation reaction proceeded for 24 h at room temperature (25 °C). TMAOH was then washed out through a series of cycles involving centrifugation at 10,000 rpm (12,850 rcf) for 10 min, discarding the supernatant, and redispersing the sediment with DI water. This process was repeated until the supernatant had a pH < 8. From here, the sediment was redispersed in DI water, shaken for 30 min, and then centrifuged at 3500 rpm (2550 rcf) for 30 min. The resulting black supernatant was carefully decanted to collect the delaminated MXene.

To obtain free-standing films, the delaminated MXene flake colloid was filtered via vacuum-assisted filtration through a porous membrane (Celgard 3501, 64 nm pore size, polypropylene). The resulting films were separated from Celgard and stored in a vacuum desiccator at room temperature.

### Structural Characterization

Rigaku SmartLab (40 kV/30 mA) and MiniFlex (40 kV/15 mA) X-ray diffractometers were used with Ni-filtered Cu K_α_ radiation. The step size of the scan was 0.01°, with a step duration of 4 s for MAX-phase powders and 2 s for films. Rietveld refinement of the MAX phases was done with the GSAS II Python code.

Scanning transmission electron microscopy (STEM) and energy dispersive X-ray spectroscopy (EDS) measurements were conducted using two aberration-corrected microscopes from Thermo Fisher Scientific (USA), the Argonne PicoProbe Analytical Electron Microscope (AEM)^50^ [A] and the Titan Low-Base microscope. All these works have been performed at 300 kV employing high-angle annular dark field (HAADF)-STEM imaging. EDS was developed using the ANL XPAD system, having access to its ultrasensitivity 4.5 sR detector. During STEM-EDS acquisitions, the electron beam current on the samples was <110 pA.

All SIMS experiments were performed on the CAMECA IMS SC Ultra instrument with cesium ions as primary ions. To reach the atomic depth resolution, a series of modifications of the measurement procedure was applied, which included high incident angle bombardment (75°), ultralow impact energy (100 eV), in situ ion polishing, optimization of extraction parameters, super cycle, and advanced beam positioning. The details of each concept were presented in the previous article.^51^ Deconvolution and calibration protocols were applied to quantify the results and determine the exact composition of each atomic layer with ±1% precision.^52^

### Electrochemistry

For electrochemical characterization, PFA Swagelok cells were used to construct three-electrode cells. The current collector was a glassy carbon electrode, while the free-standing MXene films were used as the working electrodes (electrode surface area 7.060 mm^2^). 95 wt % Activated carbon (YP-50) and 5 wt % polytetrafluoroethylene (PTFE) were used to construct the counter electrode. The separator was a Celgard 3501. The reference electrode used was Hg/Hg_2_SO_4_ in saturated K_2_SO_4_. Three M H_2_SO_4_ (degassed with Argon, 1 h) was used as the electrolyte for all electrochemical measurements. All electrochemical cells were precycled at 20 mV s^–1^ for 100 cycles before testing. Specific capacitance was calculated from the anodic CV scans according to
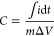
where *C* is the specific capacitance, *i* is the measured current as a function of time (*t*), m is the mass of the working electrode, and Δ*V* is the potential window.

### Optical Properties and Stability

UV–vis–NIR spectra were collected using a Thermo Scientific Evolution 201 spectrometer in transmission mode from 200 to 1000 nm using a blank cuvette with DI water as the background. UV–vis–NIR was conducted from 200 to 1000 nm with an integration time of 1 s (Evolution 201, Thermo Fisher Scientific, USA). When the change in absorbance over time was quantified, the absorbance values were extracted from the wavelengths marked in Figure S9.

### Magnetotransport Measurements

The resistivity and MR behavior of the films were measured with a Quantum Design EverCool II physical property measurement system (PPMS). For all measurements, sections of the freestanding MXene films were wired into a four-point probe geometry with silver paint, and the data was recorded from 10 to 300 K at <5 Torr helium pressure. Resistivity (ρ) was calculated via the following equation:
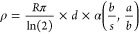
where *R* is the resistance (in Ohms), *d* is the sample thickness (in cm), and α is a geometric correction factor (where *a* and *b* are the length and width of the rectangular film, respectively, and *s* is the distance between the probes). The electrical conductivity of the films was measured both with a four-point probe (ResTest, Jandel Engineering Ltd., Bedfordshire, U.K., probe distance 1 mm, average of 5 points across the film) and with the PPMS system. Magnetoresistance (MR) measurements were performed with the magnetic field applied out-of-plane to the MXene films. MR is defined as
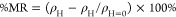
where ρ_H_ and ρ_H=0_ are the resistivities in the presence and absence of the applied magnetic field (*H*), respectively.

## Results and Discussion

### Synthesis of the (Mo_2/3_V_1/3_)_*n*+1_C_*n*_ System

**Figure 1:**
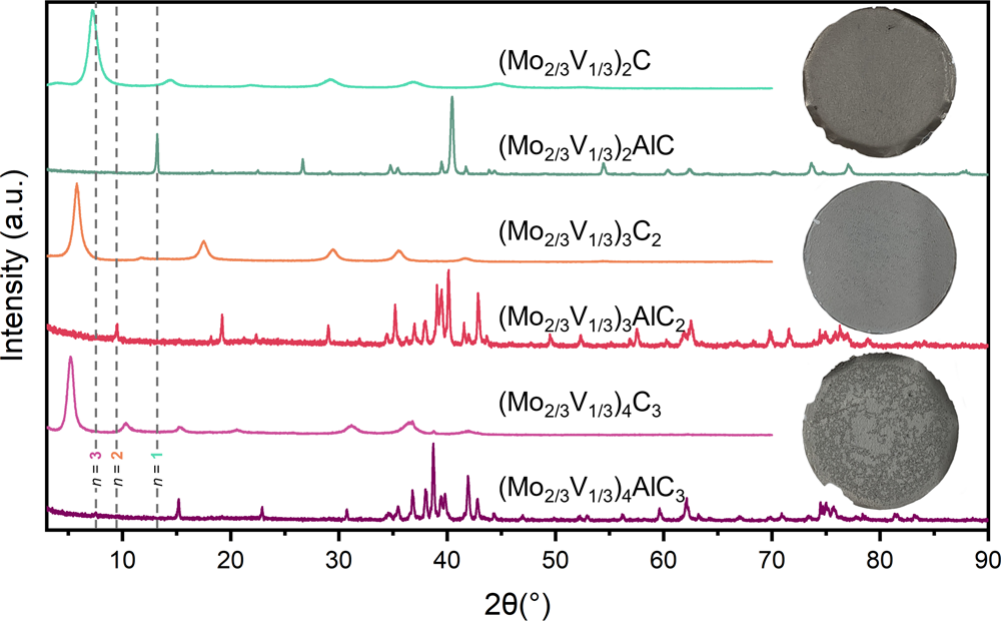
X-ray diffraction (XRD) patterns of the phase pure (Mo_2/3_V_1/3_)_*n*+1_AlC_*n*_ MAX phases and the single-layer (Mo_2/3_V_1/3_)_*n*+1_C_*n*_ MXene family. For the MAX phase patterns, the dashed line aligns with the (002) peak of the MAX phase for *n* = 1, 2, and 3, showing that the (002) of each MAX phase does not overlap with the other patterns. Inset pictures show the delaminated films corresponding to each MXene (Ø = 47 mm).

To synthesize a family of MAX phases with the composition (Mo_2/3_V_1/3_)_*n*+1_AlC_*n*_, the Mo:V ratio was held constant at Mo:V = 2/3:1/3 based on the previously reported (Mo_2/3_V_1/3_)_4_AlC_3_ MAX phase.^38^ While (Mo_2/3_V_1/3_)_3_AlC_2_ has been previously reported, it has yet to be synthesized as a pure MAX phase.^37,53^ Herein, the addition of a small amount of V_2_O_3_ promotes the synthesis of (Mo_2/3_V_1/3_)_3_AlC_2_ free of other MAX phase impurities. All synthesized MAX phases were HCl-washed in 9 M HCl (Figure S1) and analyzed via XRD as a confirmation of synthesis (Figure 1). Through Rietveld refinement (Figure S2), the lattice parameters of each MAX phase were determined. For (Mo_2/3_V_1/3_)_2_AlC, the *a*-lattice parameter was 2.97 Å with a *c*-lattice of 13.34 Å. For (Mo_2/3_V_1/3_)_3_AlC_2_, the *a*-lattice parameter was also 2.97 Å with a *c*-lattice of 18.40 Å. For (Mo_2/3_V_1/3_)_4_AlC_3_, the *a*-lattice parameter was 2.99 Å with a *c*-lattice of 23.21 Å. There is a clear dependency of the *c-*lattice on *n*, as increasing *n* results in an increase in the *c*-lattice parameter by ∼5 Å per integer value of *n*. This trend is observed in other MAX phases as well; for example, Ti_2_AlC (13.4 Å) and Ti_3_AlC_2_ (18.4 Å); Nb_2_AlC (13.9 Å) and Nb_4_AlC_3_ (24.0 Å).^25^ However, the *a*-lattice parameter does not exhibit this dependence on *n*. Apart from the MAX phases, intermetallic and carbide impurities were present in (Mo_2/3_V_1/3_)_2_AlC and (Mo_2/3_V_1/3_)_3_AlC_2_, and to a lesser extent (Mo_2/3_V_1/3_)_4_AlC_3_ (Figure S2). Notably, the relative intensity of the (002) peak in the *n* = 1 sample is much greater than that of the *n* = 2 and 3 samples. This is common in Mo-based MXenes, where the low atomic scattering factor of Mo, in combination with the ordered structure, results in a lower intensity of the characteristic (002) peak.^30,38^

As a result of the removal of the A-layer during etching and subsequent delamination of the flakes, the (002) peak of the MAX phases shifts to lower 2θ to indicate the expansion of the *c*-lattice parameter. As with the MAX phases, the *c*-lattice parameter increases by approximately 5 Å as *n* increases. The broadening of the peaks is the result of the semirandom alignment of the flakes in the free-standing film. The lack of MAX phase peaks in the film XRD patterns indicates successful conversion to the MXene phase from the MAX phase precursors during synthesis. As typical with MXene XRD patterns, only the (00*l*) peaks are present. While MAX phases show an increased number of (002) reflections as *n* increases, this trend does not necessarily apply to the delaminated MXene films due to the in-plane alignment of the flakes.^16^

### Structural Analysis of the (Mo_2/3_V_1/3_)_*n*+1_C_*n*_ System

**Figure 2:**
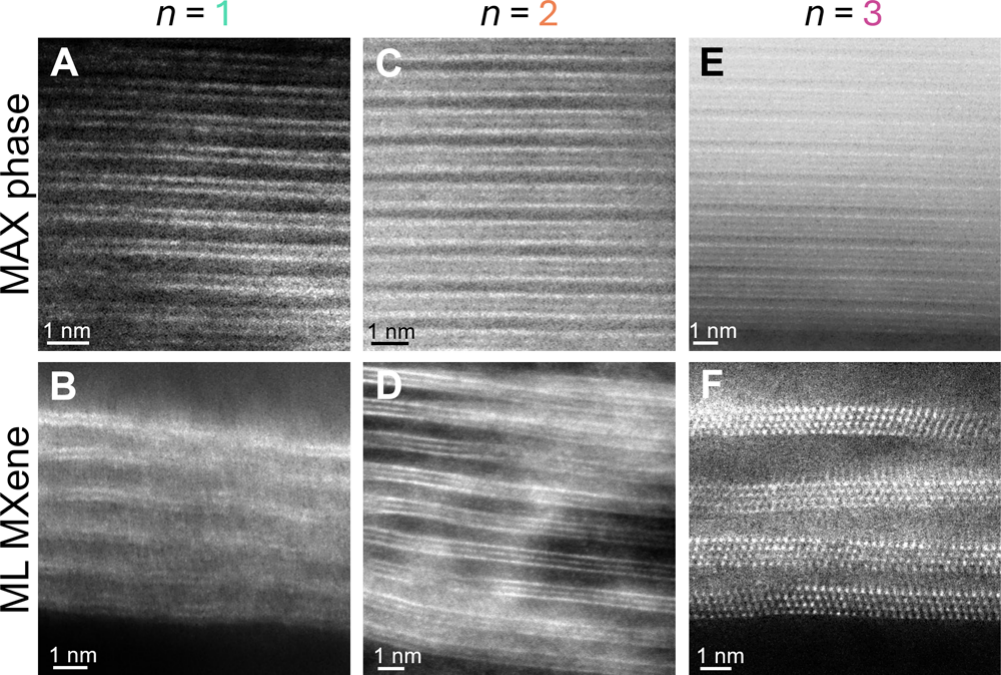
High-angle annular dark-field scanning transmission electron microscopy (HAADF-STEM) images of the (Mo_2/3_V_1/3_)_*n*+1_C_*n*_ system showing cross-sectional images of the MAX phases and Multilayer (ML) MXenes. (A) (Mo_2/3_V_1/3_)_2_AlC MAX phase and (B) ML (Mo_2/3_V_1/3_)_2_C MXene showing two layers of transition metals and the disappearance of the A-layer after etching. (C) (Mo_2/3_V_1/3_)_3_AlC_2_ MAX phase and (D) ML (Mo_2/3_V_1/3_)_3_C_2_ MXene show evidence of atomic ordering due to the brighter outer layers of the MXene unit. (E) (Mo_2/3_V_1/3_)_4_AlC_3_ MAX phase and (F) ML (Mo_2/3_V_1/3_)_4_C_3_ MXene.

Microscopic analysis of the (Mo_2/3_V_1/3_)_*n*+1_C_*n*_ system was conducted via HRSTEM imaging to confirm the number of layers associated with each MAX phase and corresponding MXene. As can be seen in Figure 2, the MAX phases exhibit uniform layering of the MXene unit cell sandwiched by the A-layer, confirming the M_*n*+1_AX_*n*_ structure characteristic of MAX phases. After etching, the A-layer is removed, resulting in separated MXene layers, some of which exhibit slight buckling. Interestingly, (Mo_2/3_V_1/3_)_3_C_2_ exhibits evidence of out-of-plane ordering of Mo and V observable as a difference in brightness between the outside and inside layers on the MXene unit cell. STEM energy-dispersive X-ray Spectroscopy (EDS) was utilized to investigate the ordering within the lattices, but electron beam damage limitations prevented accurate determination of ordering (Figures S3–S8 and Table S1).

**Figure 3:**
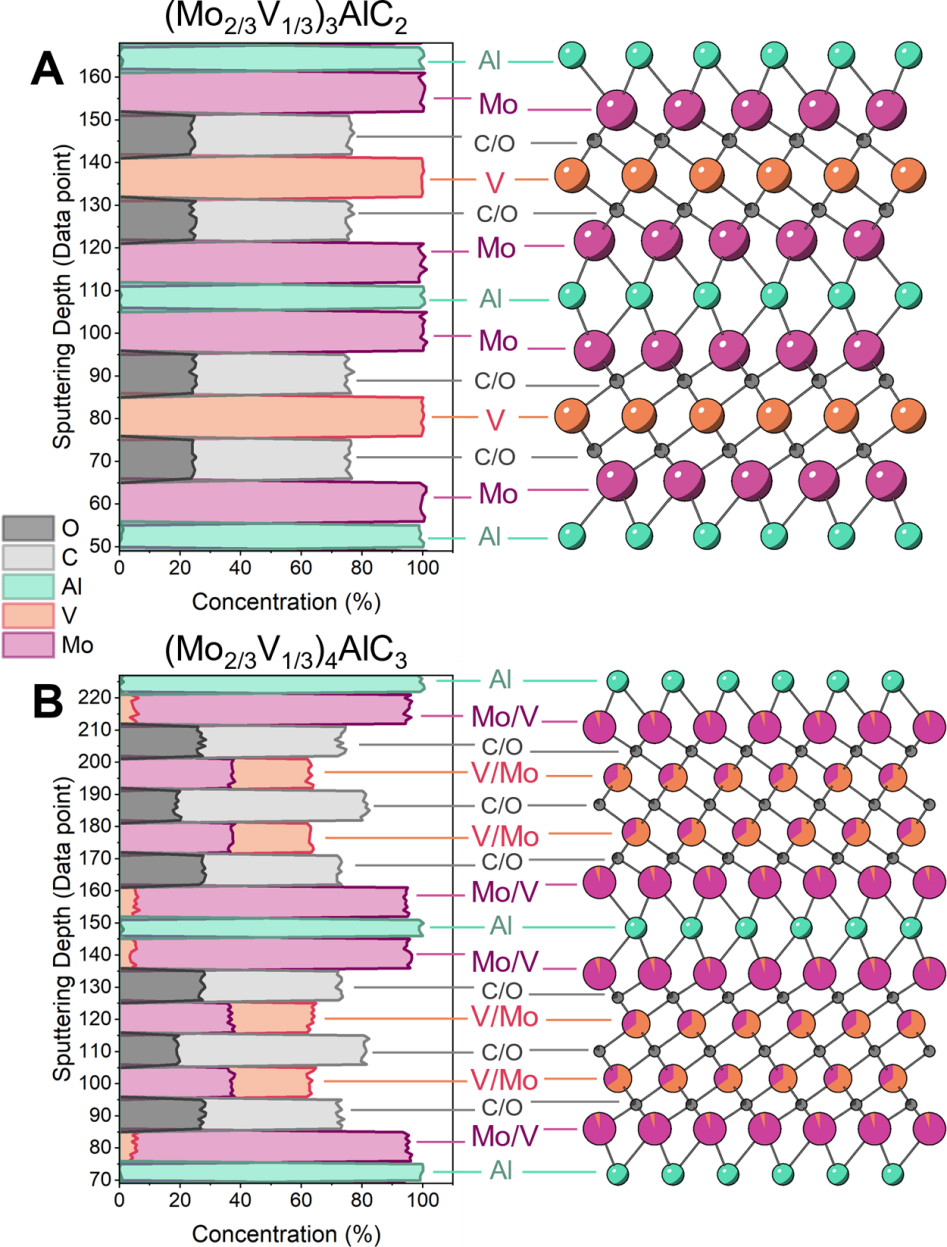
Depth profiles of atomic concentrations obtained by secondary ion mass spectrometry (SIMS) measurements of (A) (Mo_2/3_V_1/3_)_3_AlC_2_ and (B) (Mo_2/3_V_1/3_)_4_AlC_3_ with accompanying crystal structures. The atoms in the structures are colored according to the concentration of each element measured via SIMS.

To confirm the possibility of Mo and V ordering within the surface and subsurface metal layers, secondary ion mass spectrometry (SIMS) was performed on (Mo_2/3_V_1/3_)_3_AlC_2_ and (Mo_2/3_V_1/3_)_4_AlC_3_ but not (Mo_2/3_V_1/3_)_2_AlC since the latter does not have subsurface metal layers. Confirming the results in Figure 2, (Mo_2/3_V_1/3_)_3_AlC_2_ displays ordering on the M-site, with Mo occupying the outer layers and V in the inner layer (Figure 3A). Interestingly, (Mo_2/3_V_1/3_)_4_AlC_3_ exhibits preferential site occupancy also, with Mo residing primarily in the outer layers and V in the inner layers (Figure 3B). The presence of order in this system is likely due to the large size difference between Mo and V, which results in the favorable separation of the atoms.^54^ The SIMS analysis also reveals that (Mo_2/3_V_1/3_)_3_AlC_2_ and (Mo_2/3_V_1/3_)_4_AlC_3_ are both oxycarbides with ∼20% of the X-site occupied by oxygen (Table S2). While many MAX phases (and MXenes) are oxycarbides due to unoptimized synthesis, the oxycarbide nature of these MAX phases may be attributed to the use of vanadium powders during synthesis, which has the propensity to oxidize.^51^ While this SIMS analysis focuses on the MAX phase, further studies of this system should extend the analysis to the MXene composition including identification of the surface terminations.

### Optical Properties and Stability

**Figure 4:**
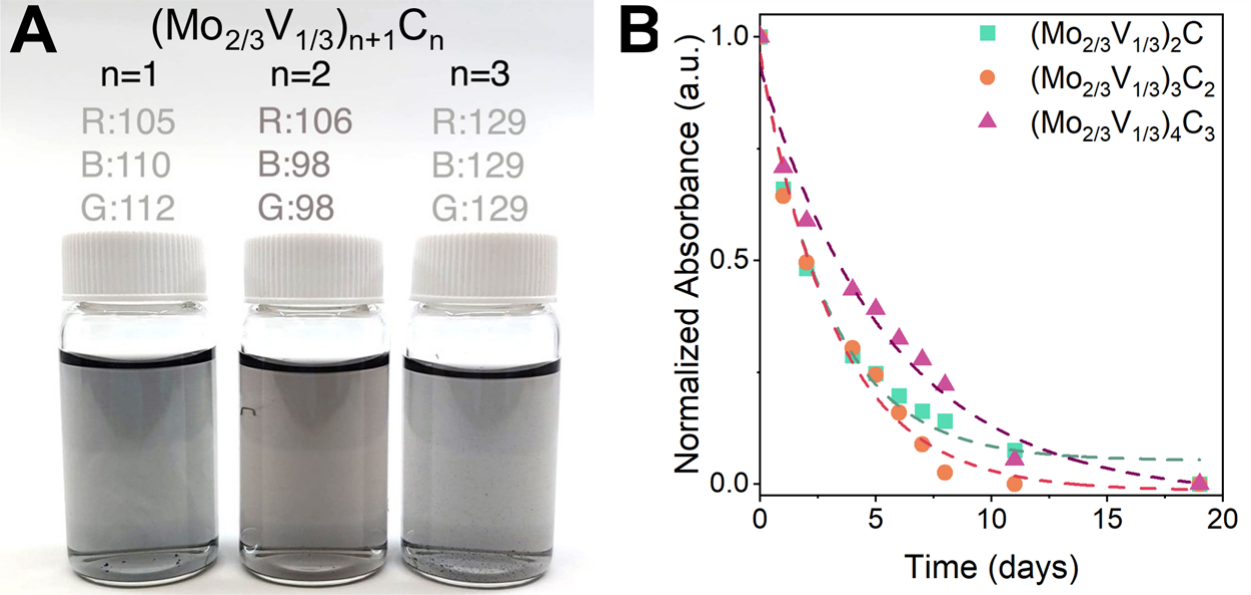
Accelerated stability measurements of the (Mo_2/3_V_1/3_)_*n*+1_C_*n*_ system. (A) Single-layer MXene suspended in water for stability measurements. (B) Solution stability of the MXene-in-water solutions. The dashed lines represent the fitted exponential decay function *f*(*x*) = *y*_0_ + *A*e^*–x*/τ^ where *y*_0_ is the offset value, *A* is the amplitude, and τ is the time constant. The samples were stored in air at ambient temperature in the dark.

The stability of MXenes is a highly researched topic, with studies attempting to understand the mechanisms behind MXene hydrolysis and oxidation,^55^ or to improve MXene’s colloidal stability through improvements in synthesis techniques.^56^ While many techniques, such as chromatography, can be used to track the decomposition process, UV–vis spectroscopy is most commonly used, providing an ability to compare between MXenes of different compositions.^57^ To monitor the decomposition behavior of the (Mo_2/3_V_1/3_)_*n*+1_C_*n*_ MXenes over time, dilute single-layer solutions in water were stored at ambient temperature in air, and their absorbance was measured over time in accelerated decomposition studies (Figure 4). Consistently, the (Mo_2/3_V_1/3_)_*n*+1_C_*n*_ system exhibits one broad extinction peak in the UV–vis–NIR spectrum that appears to shift to lower wavelengths with increasing *n*, though further studies are required to properly interpret the spectrum (Figure S9). The stability was quantified by fitting the change in absorbance with an exponential decay function; from these fittings, the time constant (τ) of decay can be extracted, which for (Mo_2/3_V_1/3_)_2_C is 3.0 days, for (Mo_2/3_V_1/3_)_3_C_2_ is 3.3 days, and for (Mo_2/3_V_1/3_)_4_C_3_ is 5.6 days. This data establishes a trend of higher *n* MXenes being more stable than their counterparts.^2,58^ This trend is seen in other MXenes, as Ti_3_C_2_ is more stable than Ti_2_C, and Nb_4_C_3_ is more stable than Nb_2_C.^41,42,55^ Therefore, it is likely that increasing *n* results in increased colloidal stability of the MXene.

### Electronic Transport

**Figure 5:**
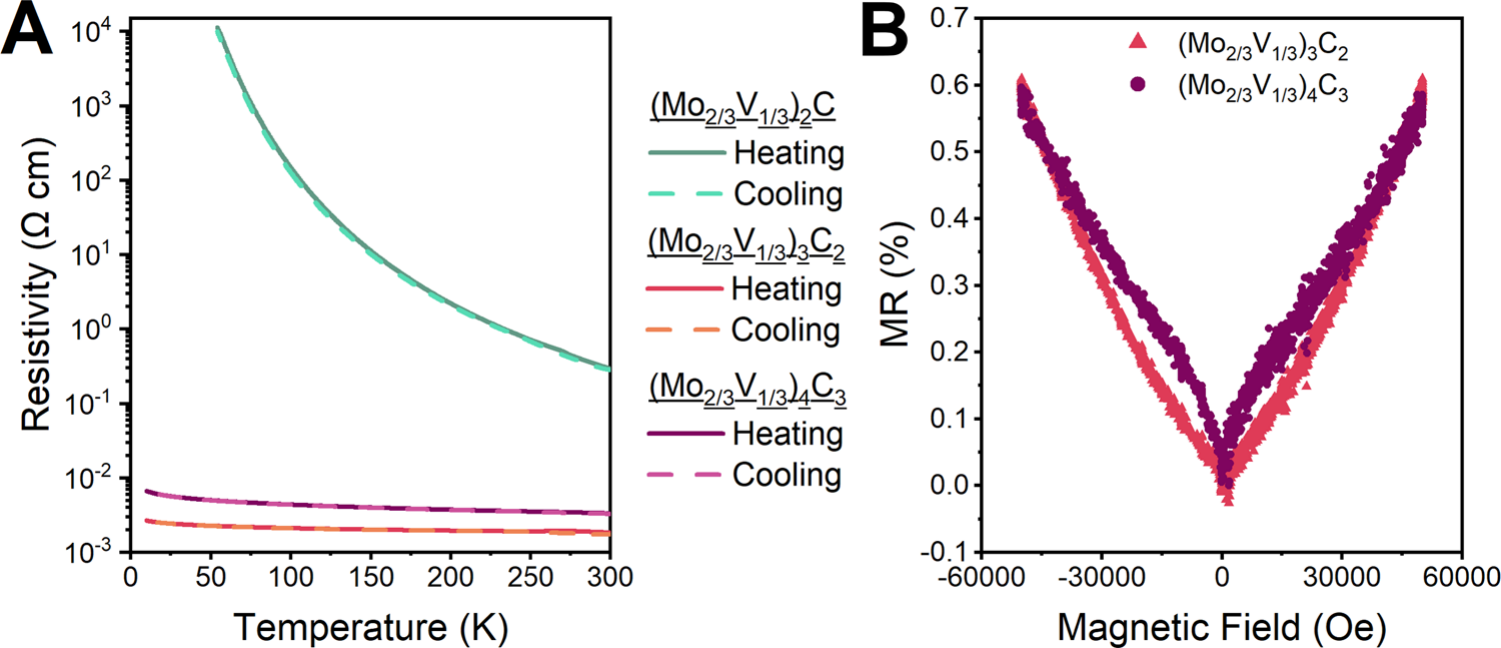
Electronic properties of the (Mo_2/3_V_1/3_)_*n*+1_C_*n*_ system. Resistivity (ρ) versus Temperature behavior of (A) all (Mo_2/3_V_1/3_)_*n*+1_C_*n*_ MXenes plotted on the log scale. The thermal hysteresis loop present after 250 K can be attributed to intercalated water. (B) Magnetoresistance at 10 K of (Mo_2/3_V_1/3_)_3_C_2_ and (Mo_2/3_V_1/3_)_4_C_3_. Although measurements were attempted, no data at 10 K could be obtained from (Mo_2/3_V_1/3_)_2_C as it was too insulating.

The (Mo_2/3_V_1/3_)_*n*+1_C_*n*_ family provides a unique opportunity to directly investigate the role of *n* in macroscopic electronic transport. As seen in Figure 5A, the transport behavior of the *n* = 1 sample differs significantly from that of the *n* = 2 and 3 materials. (Mo_2/3_V_1/3_)_3_C_2_ and (Mo_2/3_V_1/3_)_4_C_3_ exhibit resistivity that is orders of magnitude less than (Mo_2/3_V_1/3_)_2_C (Figure S10). This results in (Mo_2/3_V_1/3_)_2_C having the lowest conductivity of the three measured MXenes (3.5 S cm^–1^, compared to 490 S cm^–1^ for (Mo_2/3_V_1/3_)_3_C_2_, and 300 S cm^–1^ for (Mo_2/3_V_1/3_)_4_C_3_, measured at 300 K; see Table S3). Strikingly, the resistivity of (Mo_2/3_V_1/3_)_3_C_2_ and (Mo_2/3_V_1/3_)_4_C_3_ displays a minimal temperature dependence behavior, which is typical for disordered metals. In contrast, the resistivity of (Mo_2/3_V_1/3_)_2_C increases by over a factor of 10^4^ on cooling from 300 to 50 K. This behavior is consistent with previous reports of Mo-based MXenes. In Mo_1.33_C, a dρ/d*T* < 0 spanning orders of magnitude of resistivity was observed, while in Mo_2_TiC_2_ and Mo_2_Ti_2_C_3_, behavior similar to our *n* = 2 and 3 samples was reported.^59,60^ The general consistency of the transport trends as a function of *n* highlights the importance of having a subsurface M-site layer for maintaining low resistivity (<0.01 Ω cm) down to low temperatures. It is likely that the structure of M_2_X MXenes results in lower conductivity because all metal layers are bound to strongly electronegative surface functional groups (−O and −F) that decrease the contribution from metal orbitals to the density of states near the Fermi edge.^44^ This trend is also observed in Ti_2_C (∼1600 S cm^–1^) vs Ti_3_C_2_ (>20,000 S cm^–1^), Nb_2_C (∼5 S cm^–1^) vs Nb_4_C_3_ (∼75 S cm^–1^), and V_2_C (∼1250 S cm^–1^) vs V_4_C_3_ (1350 S cm^–1^).^25,61,62^

To further investigate the transport mechanisms, the magnetoresistance (MR) of the samples was measured at 10 K. As seen from Figure 5B, the measured MXenes have positive MR dependence, common to Mo-MXenes but in contrast to Ti_3_C_2_ and Cr_2_TiC_2_, which exhibit a negative dependence.^37,63,64^ However, the positive MR measured in (Mo_2/3_V_1/3_)_3_C_2_ and (Mo_2/3_V_1/3_)_4_C_3_ does not follow a simple *H*^2^ dependence as expected for nonmagnetic metals. Given that metallic MXenes derived largely from 3d transition metals exhibit negative MR while 4d-derived MXenes exhibit positive MR, we speculate that spin–orbit coupling may play a central role in the low-temperature magnetotransport behavior, as has previously been established in ultrathin metals that exhibit weak localization.^65^ For example, low-temperature positive MR in thin Mo–C films was observed and displayed excellent agreement with models based on spin–orbit scattering with an antilocalization mechanism.^66^ While further work is needed to clarify the mechanisms giving rise to the diverse magnetoresistive properties of MXenes, this study confirms that positive MR is a robust feature of Mo-based MXenes.

### Electrochemical Energy Storage

**Figure 6:**
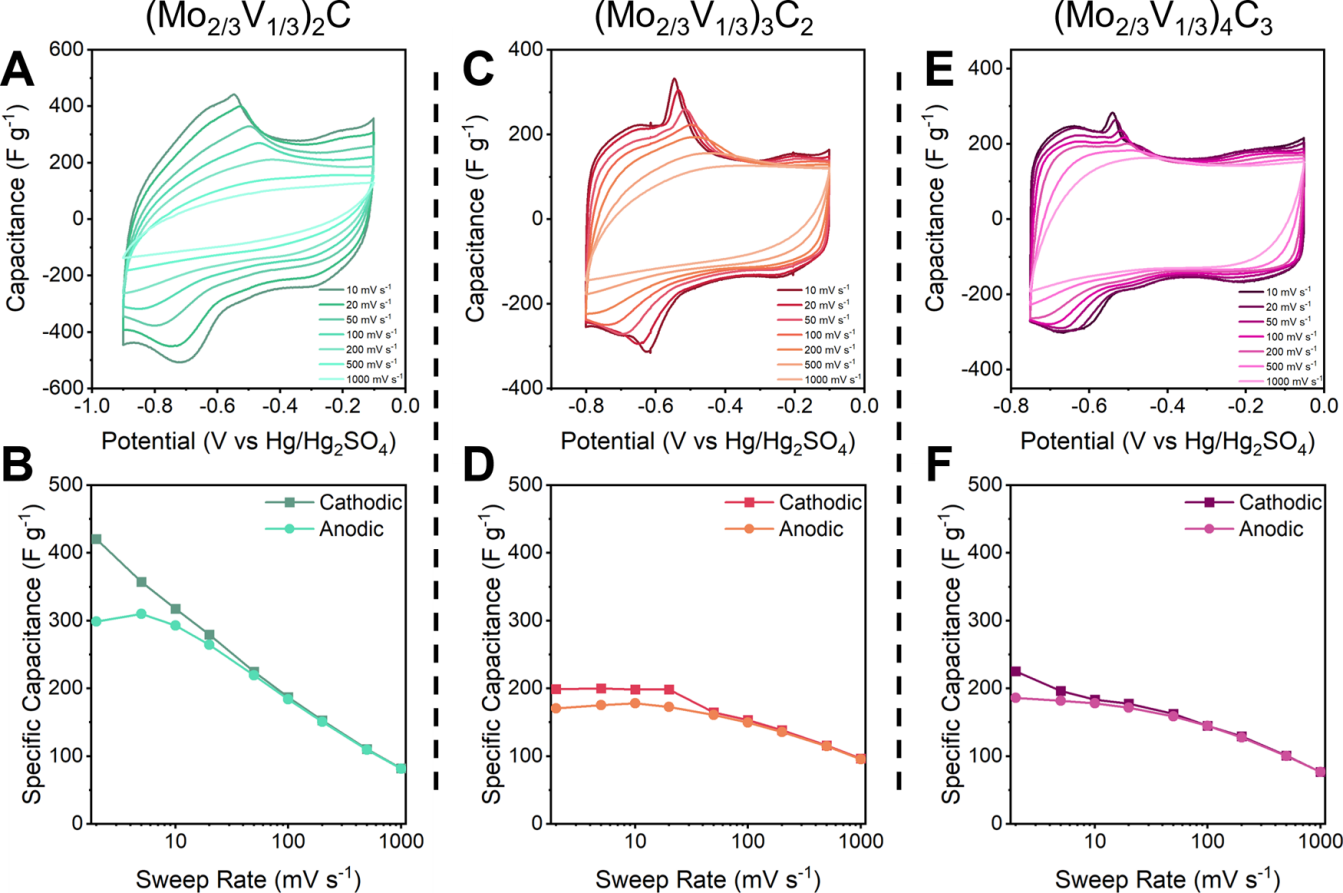
Cyclic voltammograms of (A) (Mo_2/3_V_1/3_)_2_C, (C) (Mo_2/3_V_1/3_)_3_C_2_, and (E) (Mo_2/3_V_1/3_)_4_C_3_. Specific capacitances of (B) (Mo_2/3_V_1/3_)_2_C, (D) (Mo_2/3_V_1/3_)_3_C_2_, and (F) (Mo_2/3_V_1/3_)_4_C_3_ as a function of sweep rate. For all measurements, the electrolyte was 3 M H_2_SO_4_.

The electrochemical behavior of the (Mo_2/3_V_1/3_)_*n*+1_C_*n*_ system was studied in 3 M H_2_SO_4_. As seen in Figure 6A,C,E, the CV profiles for the (Mo_2/3_V_1/3_)_*n*+1_C_*n*_ system all display small anodic and cathodic peaks with a quasi-rectangular shape. This redox couple agrees with what we observed from Mo_4_VC_4_ in 3 M H_2_SO_4_, which was attributed to pseudocapacitive redox of proton adsorption on oxygen terminations with a charge transfer at the transition metal sites.^67^ The redox peaks that are present in all the CVs shift toward negative potential as *n* decreases. Additionally, as *n* decreases, there is a shift of the electrochemical window toward negative potentials (Figure S11). (Mo_2/3_V_1/3_)_2_C shows a stable electrochemical window from −0.90 to −0.10 V vs Hg/Hg_2_SO_4_; (Mo_2/3_V_1/3_)_3_C_2_ from −0.80 to −0.10 V vs Hg/Hg_2_SO_4_; (Mo_2/3_V_1/3_)_4_C_3_ from −0.75 to −0.05 V vs Hg/Hg_2_SO_4_. (Mo_2/3_V_1/3_)_2_C has the largest and most negative behavior, likely due to a combination of M and *n* influencing factors: the M_2_X structure is essentially entirely surface, giving it the highest concentration of active surface sites. Additionally, (Mo_2/3_V_1/3_)_2_C has the most exposed vanadium of all the structures, which likely influences its behavior to act more like V_2_C with its lower and larger electrochemical window.^62^ As for (Mo_2/3_V_1/3_)_3_C_2_ and (Mo_2/3_V_1/3_)_4_C_3_, the increased hydrogen evolution catalytic behavior, as indicated by the smaller windows, may be attributed to Mo, which dominated the surface sites (Figure 3).^68^

Of the tested MXenes, (Mo_2/3_V_1/3_)_2_C has the highest specific capacitance, whereas (Mo_2/3_V_1/3_)_3_C_2_, and (Mo_2/3_V_1/3_)_4_C_3_ show similar performance (Figure 6B,D,F). This superior gravimetric performance of (Mo_2/3_V_1/3_)_2_C can likely be attributed to the higher concentration of active transition metal surface sites in the M_2_X structure compared to the larger M_3_X_2_ or M_4_X_3_ structures. However, (Mo_2/3_V_1/3_)_2_C shows the lowest rate capability of the (Mo_2/3_V_1/3_)_*n*+1_C_*n*_ system, likely due to its lower conductivity. Optimization of the synthesis procedure for (Mo_2/3_V_1/3_)_2_C may increase its overall quality, stability, and conductivity and result in a higher rate capability for this system. Overall, the performance of the (Mo_2/3_V_1/3_)_*n*+1_C_*n*_ system indicates that M_2_X MXenes are likely superior for electrochemical applications due to their increased concentrations of active sites.

## Conclusions and Outlook

Herein, we have synthesized (Mo_2/3_V_1/3_)_*n*+1_AlC_*n*_ MAX phases and successfully etched and delaminated them to (Mo_2/3_V_1/3_)_*n*+1_C_*n*_ MXenes with *n* = 1, 2, and 3. This allowed, for the first time, the study of MXene properties as a function of transition metal layers from *n* = 1 to 3. Structural analysis revealed full and partial ordering of the M-elements in (Mo_2/3_V_1/3_)_3_C_2_ and (Mo_2/3_V_1/3_)_4_C_3_, respectively, which can be attributed to the differences in the size of Mo and V. By studying their colloidal and electronic properties alongside their electrochemical behavior, trends with respect to *n* were established. The colloidal stability was found to directly increase with increasing *n*, with (Mo_2/3_V_1/3_)_4_C_3_ having much-improved stability over its lower *n* counterparts. With regard to the electronic transport behavior and electrochemical properties, it was found that the transport and electrochemical behavior of (Mo_2/3_V_1/3_)_2_C differ significantly from its higher-order counterparts. This is likely due to the lack of core layers resulting in all metal layers being bound to surface functional groups. While this supplies gravimetrically a greater concentration of active surface sites for improved electrochemical behavior, the lack of conducting core results in significantly increased resistivity. While this is only the first examination into the effects of *n*, this system of MXenes will allow for more concrete studies into the effects of *n* to allow for better tailoring of the MXene properties across M_*n*+1_X_*n*_.

## Supporting Information Available

Rietveld refinement of the MAX phase XRD patterns; local chemical composition via STEM-EDS analysis and SIMS analysis quantification; UV–vis–NIR spectrum; conductivities of the (Mo_2/3_V_1/3_)_*n*+1_C_*n*_ system measured by both the 4-point probe and the physical properties measurement (PPMS) system; and electrochemical figure replotted with identical axis (PDF)
