# Supplementary material for: Synthesis of Three Isoelemental MXenes and Their Structure–Property Relationships
Source: J Am Chem Soc. 2024 Oct 31;146(45):31159–68. doi: 10.1021/jacs.4c11111 (PMC11565644; doi:10.1021/jacs.4c11111)
Supplement: Supplementary file 1 — ja4c11111_si_001.pdf [file ja4c11111_si_001.pdf]

Supporting Information

Synthesis of Three Isoelemental MXenes and Their Structure-Property Relationships

Marley Downes[1], Christopher E. Shuck[1,2], Ruocun (John) Wang[1], Paweł Piotr Michałowski[3], Jonathan Shochat[1], Danzhen Zhang[1], Mikhail Shekhirev[1], Yizhou Yang[1], Nestor J. Zaluzec[4], Raul Arenal[5,6,7], Steven J. May[1], Yury Gogotsi[1]*

[1]A. J. Drexel Nanomaterials Institute, and Department of Materials Science and Engineering, Drexel University, Philadelphia, PA, 19104, USA

[2]Department of Chemistry and Chemical Biology, Rutgers University, Piscataway, NJ 08854, USA

[3]Łukasiewicz Research Network—Institute of Microelectronics and Photonics, 02-668, Warsaw, Poland

[4] University of Chicago, Pritzker School of Molecular Engineering, Laboratory for Energy Storage and Conversion, Chicago, and Argonne National Laboratory, Lemont, IL, 60637, USA

[5] Instituto de Nanociencia y Materiales de Aragon (INMA), CSIC-Universidad de Zaragoza, 50018 Zaragoza, Spain

[6] Laboratorio de Microscopias Avanzadas (LMA), Universidad de Zaragoza, 50018 Zaragoza, Spain

[7] ARAID Foundation, 50018 Zaragoza, Spain

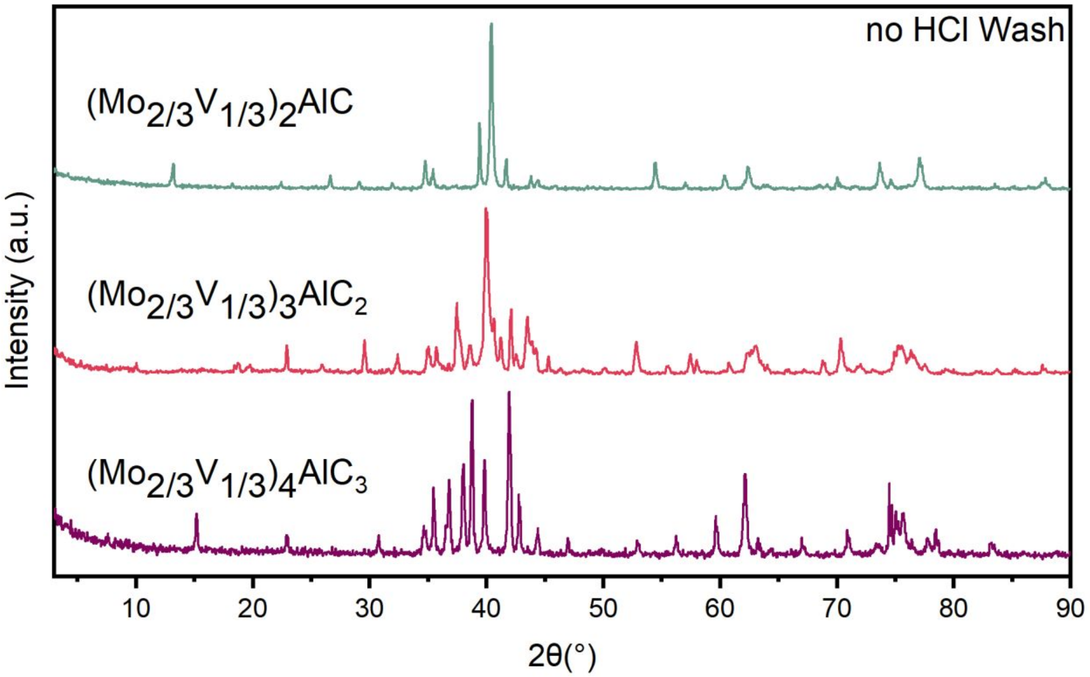

Figure S1. X-ray diffraction (XRD) patterns for the as-synthesized $(Mo_{2/3}V_{1/3})_{n+1}AlC_n$ MAX phases before being HCl-washed to remove intermetallic impurities.

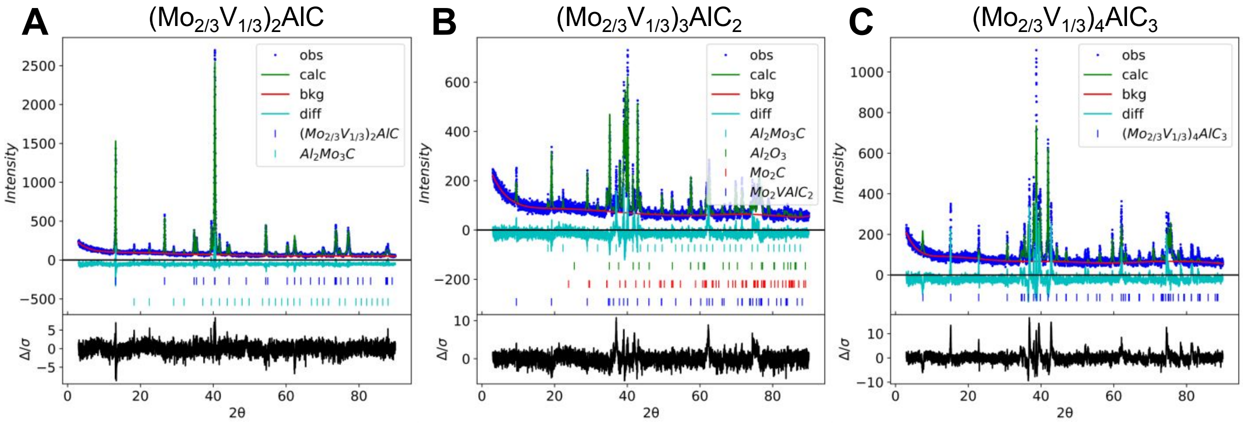

Figure S2. Rietveld refinement fittings of (A) $(Mo_{2/3}V_{1/3})_2AlC$ (R_w ~ 12.710%; 90.6 wt%) with non-MAX phase impurity Al_2Mo_3C, (B) $(Mo_{2/3}V_{1/3})_3AlC_2$ (R_w ~ 15.296%; 68.2 wt%) with non-MAX phase impurities Al_2Mo_3C, Al_2O_3, and Mo_2C, and (C) $(Mo_{2/3}V_{1/3})_4AlC_3$ (R_w ~ 22.066%) which exhibited the best fitting without accounting for possible impurities.

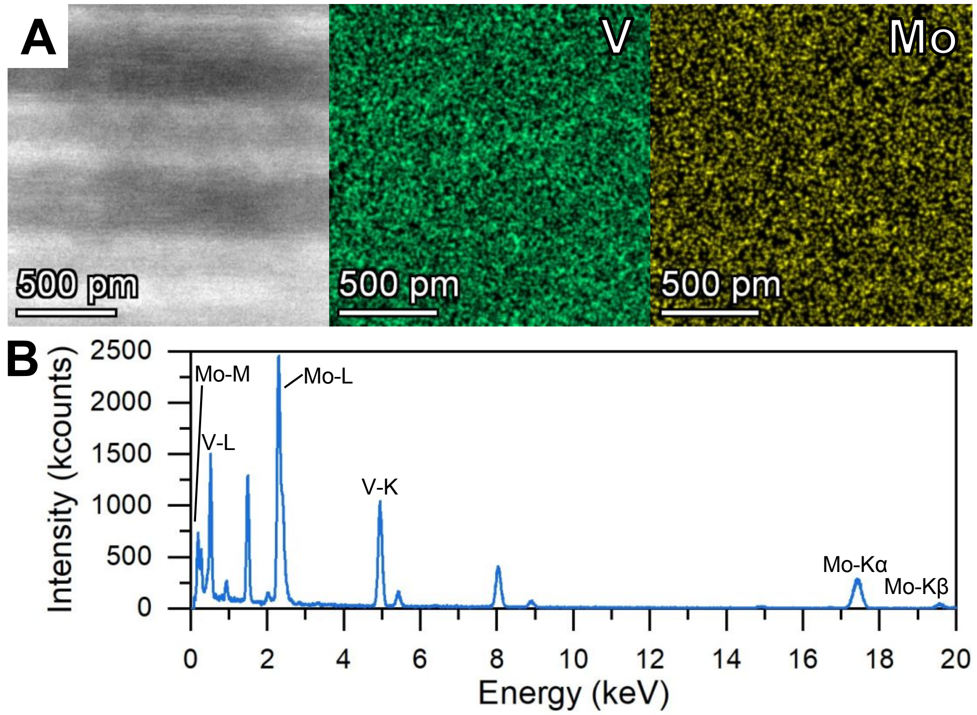

Figure S3. Chemical characterization of $(Mo_{2/3}V_{1/3})_2AlC$ MAX Phase. A) High-angle annular dark-field scanning transmission electron microscopy (HAADF-STEM) image and Energy-dispersive X-ray Spectroscopy (EDS) elemental maps of V and Mo; B) corresponding EDS Spectrum.

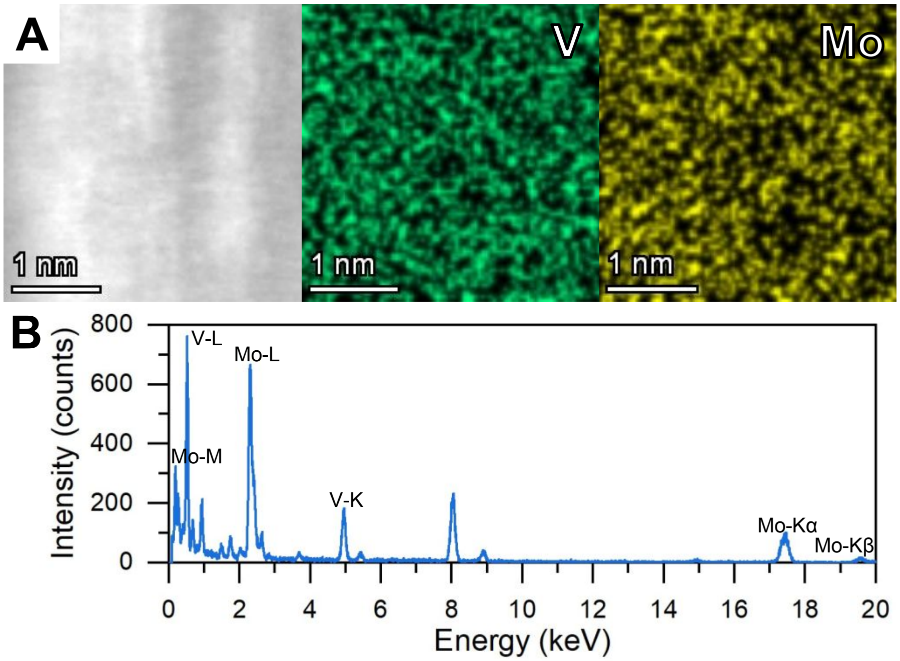

Figure S4. Chemical characterization of $(Mo_{2/3}V_{1/3})_2C$ Multilayer (ML) MXene. A) HAADF-STEM image and EDS elemental maps of V and Mo; B) corresponding EDS Spectrum.

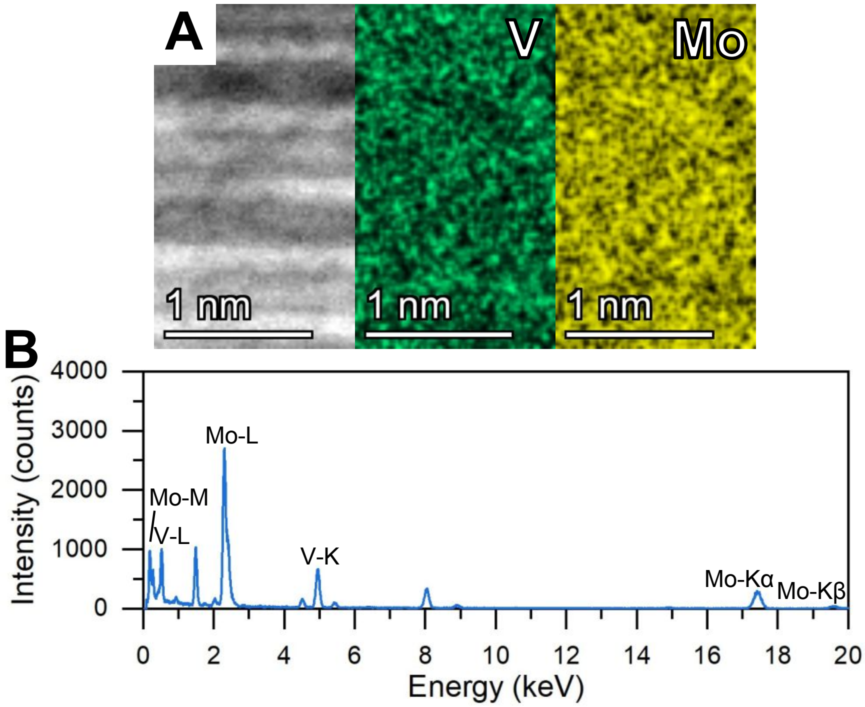

Figure S5. Chemical characterization of $(Mo_{2/3}V_{1/3})_3AlC_2$ MAX Phase. A) HAADF-STEM image and EDS elemental maps of V and Mo; B) corresponding EDS Spectrum.

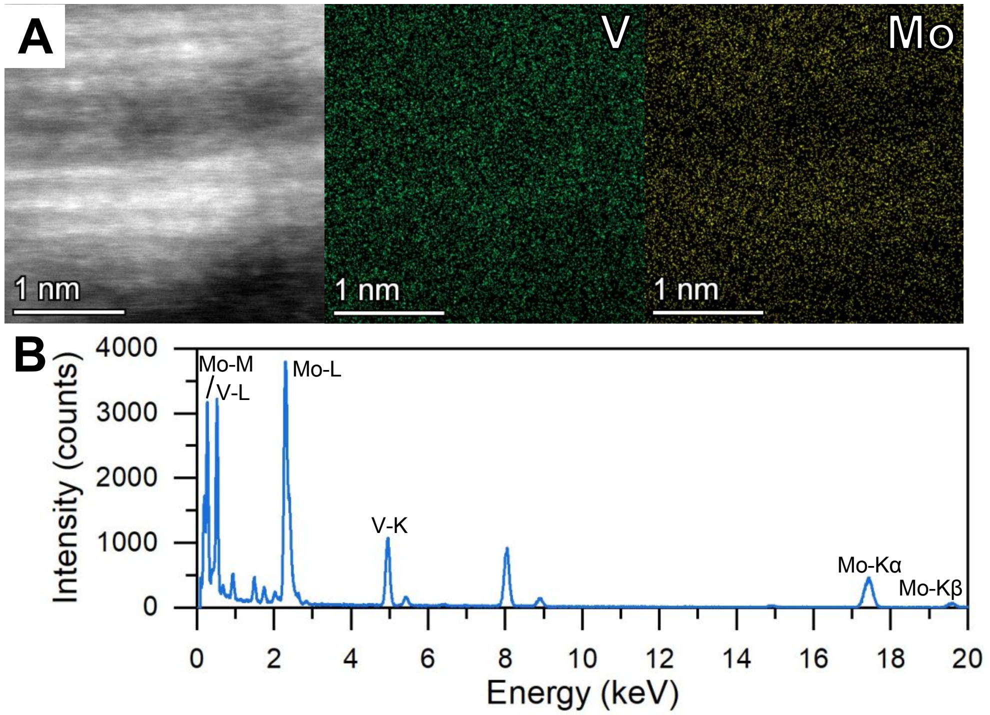

Figure S6. Chemical characterization of $(Mo_{2/3}V_{1/3})_3C_2$ ML MXene. A) HAADF-STEM image and EDS elemental maps of V and Mo; B) corresponding EDS Spectrum.

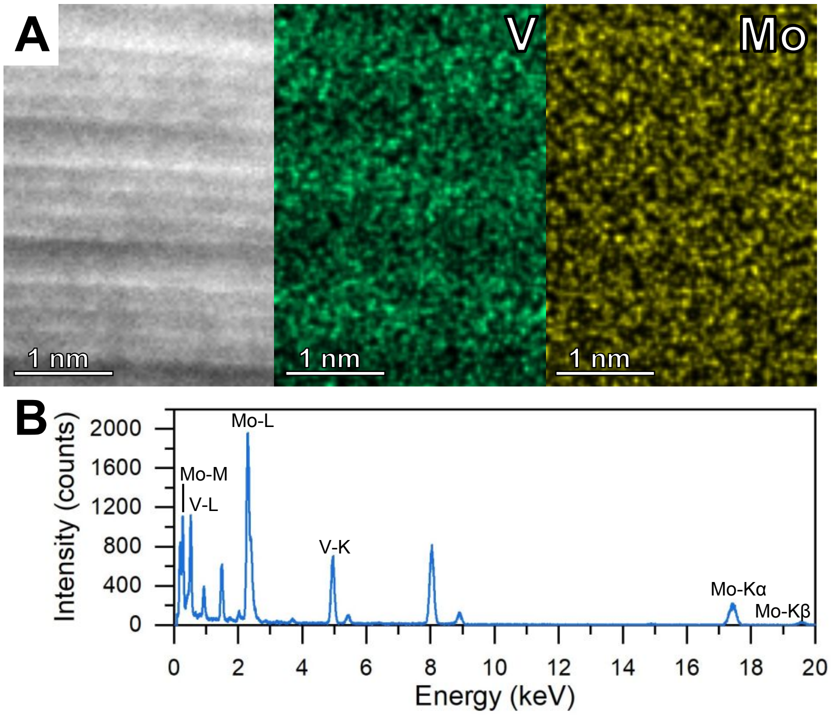

Figure S7. Chemical characterization of $(Mo_{2/3}V_{1/3})_4AlC_3$ MAX Phase. A) HAADF-STEM image and EDS elemental maps of V and Mo; B) corresponding EDS Spectrum.

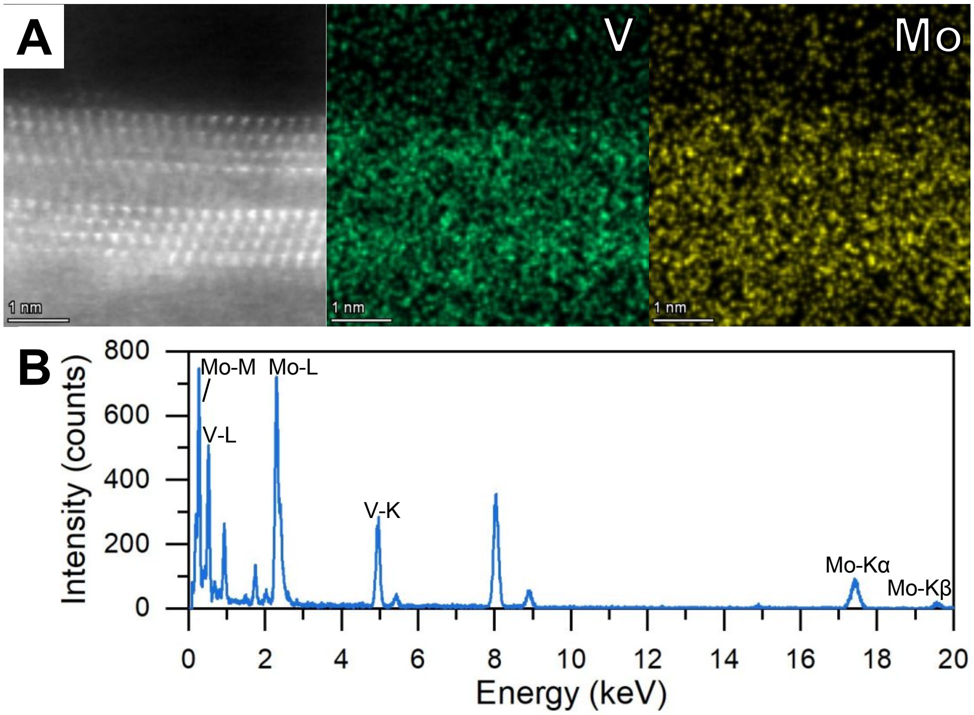

Figure S8. Chemical characterization of $(Mo_{2/3}V_{1/3})_4C_3$ ML MXene. A) HAADF-STEM image and EDS elemental maps of V and Mo; B) corresponding EDS Spectrum.

Table S1. EDS data for the concentrations of Mo and V in $(Mo_{2/3}V_{1/3})_{n+1}AlC_n$ and ML $(Mo_{2/3}V_{1/3})_{n+1}C_n$ for n = 1, 2, 3.

EDS (%)	Mo	V
***n* = 1**		
**$(Mo_{2/3}V_{1/3})_2AlC$**	67.0±2.8%	32.9±2.8%
**ML $(Mo_{2/3}V_{1/3})_2C$**	78.1±1.6%	21.9±1.6%
***n* = 2**		
**$(Mo_{2/3}V_{1/3})_3AlC_2$**	75.6±0.5%	23.7±1.4%
**ML $(Mo_{2/3}V_{1/3})_3C_2$**	73.8±5.7%	26.1±5.7%
***n* = 3**		
**$(Mo_{2/3}V_{1/3})_4AlC_3$**	67.9±2.7%	32.1±2.7%
**ML $(Mo_{2/3}V_{1/3})_4C_3$**	68.4±4.6%	31.6±4.6%

Table S2. Tabulated element concentrations for $(Mo_{2/3}V_{1/3})_3AlC_2$ and $(Mo_{2/3}V_{1/3})_4AlC_3$ calculated from SIMS data. Values are organized by layer, where M13 refers to the outer M-layers, M2 refers to the core M-layer, and X12 refers to both X-layers in the M_3AX_2 MAX phase structure. For $(Mo_{2/3}V_{1/3})_4AlC_3$, M14 refers to the outer M-layers, M23 refers to the inner M-layers, and X2 refers to the inner X-layer while X13 refers to the outer X-layers in the M_4AX_3 MAX phase structure. For both MAX phases, the composition of the Al-layer is not included.

Element	**$(Mo_{2/3}V_{1/3})_3AlC_2$**			**$(Mo_{2/3}V_{1/3})_4AlC_3$**			
	**M13**	**M2**	**X12**	**M14**	**M23**	**X2**	**X13**
**Mo**	100.1±1.2	0	0	94.9±0.6	37.0±0.6	0	0
**V**	0	100.0±1.2	0	5.0±0.6	63.0±0.6	0	0
**C**	0	0	76.0±0.6	0	0	82.0±0.5	74.0±0.6
**O**	0	0	24.0±0.6	0	0	18.0±0.6	26.0±0.6

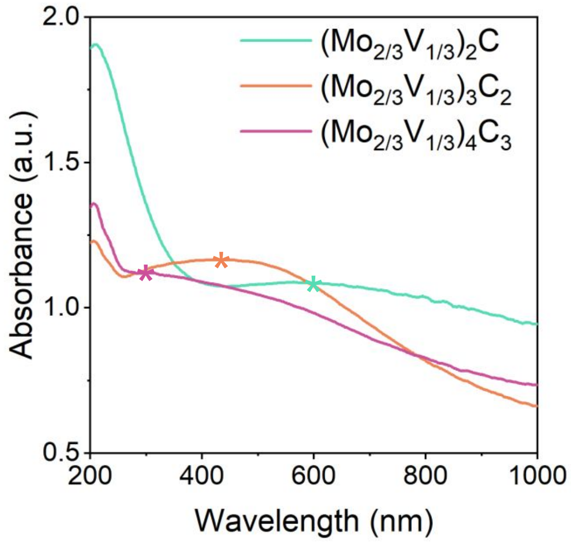

Figure S9. UV-vis-NIR spectra for single-layer $(Mo_{2/3}V_{1/3})_{n+1}C_n$ in water. Asterisks (*) represent the wavelengths where colloidal stability absorbance measurements were taken for each sample, corresponding to Figure 4.

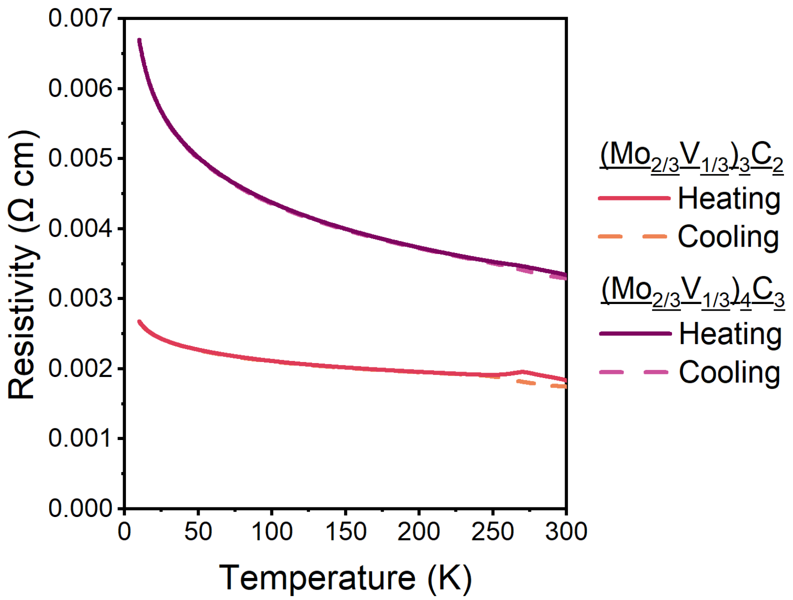

Figure S10. Resistivity (ρ) vs Temperature behavior of $(Mo_{2/3}V_{1/3})_3C_2$ and $(Mo_{2/3}V_{1/3})_4C_3$ from 10 K to 300 K. The small thermal hysteresis loop present after 250 K can be attributed to intercalated water.

Table S3. Measured electronic conductivities of the $(Mo_{2/3}V_{1/3})_{n+1}C_n$ system, showing good agreement between values measured by the 4-point probe and the physical property measurement system (PPMS) system.

$(Mo_{2/3}V_{1/3})_{n+1}C_n$	Conductivity (S cm^{-1})	
	4-point probe	PPMS (@ 300 K)
n = 1	2.5 ± 0.3	3.5
n = 2	330 ± 80	490
n = 3	280 ± 30	300

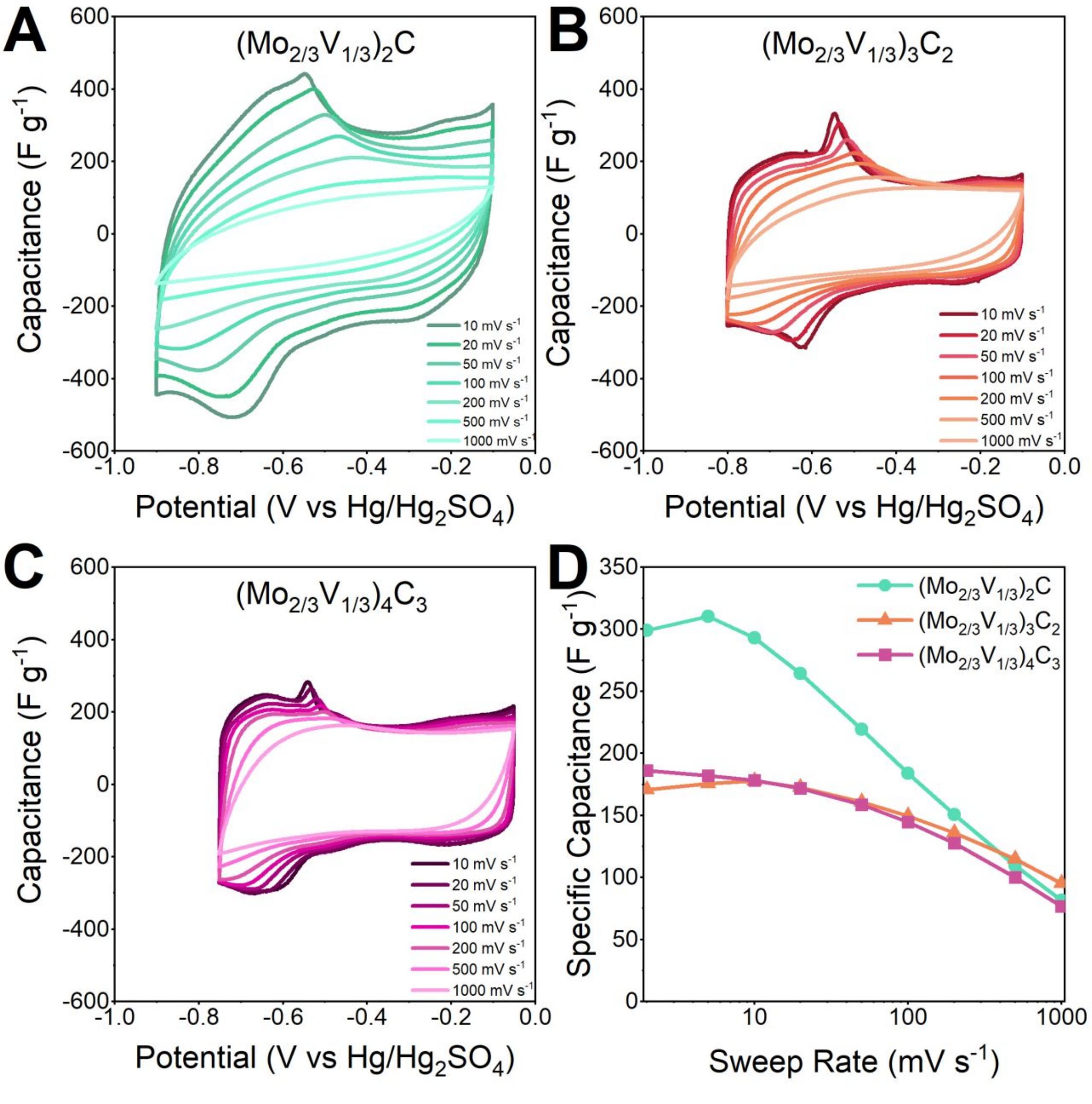

Figure S11. Replication of Figure 6, where the axes of all the CVs are identical for more accurate comparison. Cyclic voltammograms of (A) $(Mo_{2/3}V_{1/3})_2C$, (B) $(Mo_{2/3}V_{1/3})_3C_2$, and (C) $(Mo_{2/3}V_{1/3})_4C_3$. For all measurements, the electrolyte was 3 M H_2SO_4. (D) The specific capacitance of the $(Mo_{2/3}V_{1/3})_{n+1}C_n$ electrodes is plotted together for direct comparison.
